# Paradoxical embolism, deep vein thrombosis, pulmonary embolism in a patient with patent foramen ovale: a case report

**DOI:** 10.1186/1752-1947-1-104

**Published:** 2007-09-25

**Authors:** Shan Guo, Ingram Roberts, Jose Missri

**Affiliations:** 1 Department of Medicine, St. Vincent's Medical Center, Bridgeport, CT 06606, USA

## Abstract

**Introduction:**

Coexistence of pulmonary embolism and systemic arterial embolism suggest the diagnosis of paradoxical embolism which suggests the presence of intracardiac defects such as patent foramen ovale (PFO).

**Case presentation:**

A 42 year old man was found to have a paradoxical embolism in the systemic arterial circulation, in the setting of pulmonary embolism and deep vein thrombosis (DVT) in the lower extremities.

**Conclusion:**

Paradoxical embolism and intracardiac shunt should be immediately considered in a patient with pulmonary embolism and systemic arterial embolism. Diagnostic modalities included arteriogram and saline contrast echocardiography. Closure of intracardiac shunt is needed for patients who are at risk for recurrent embolic events.

## Introduction

Coexistence of pulmonary embolism and systemic arterial embolism indicates the presence of paradoxical embolism, which suggests a diagnosis of an intracardiac defect. The most common intracardiac defect associated with paradoxical embolism is patent foramen ovale (PFO), which has been described in 25–30 percent of individuals [1]. Most patients with a patent foramen ovale (PFO) remain asymptomatic. Under normal physiological conditions, patent foramen ovale (PFO) allows a small amount of L-R shunt without causing significant hemodynamic change. However, in the setting of increased right atrial pressure, significant right to left shunt can occur and lead to paradoxical embolism. The most important potential clinical manifestation due to a paradoxical embolism is ischemic stroke. There have been few reports of paradoxical emboli in systemic circulation coexistent with PE, deep vein thrombosis (DVT) and hypercoagulable state.

The most frequently cited criteria for the diagnosis of paradoxical embolism are:

1). Embolism in arterial system that is not originated from left heart or from the arterial system itself. 2). Abnormal communication between the arterial and venous systems as evidenced by imaging tests. 3). Presence of venous thrombosis or embolism in the form of deep vein thrombosis or pulmonary embolism. 4). Increased right sided pressure which contributes right to left shunting, be it transient or longstanding [2].

A case of DVT, resultant subacute PE leading to pulmonary hypertension, right to left shunt via patent foramen ovale (PFO) causing paradoxical embolus in the aorta is described. The patient was successfully treated with emergent thrombectomy of aorta, IVC filter placement and anticoagulation.

## Case presentation

A 42 year-old man with past medical history of PE was brought to the ER 30 minutes after he had sudden onset of "numbness" and "tingling sensation", accompanied by leg and groin pain. He was unable to stand, which was followed by complete loss of sensation in both legs with cyanosis. In addition to his symptoms in lower extremities, he also complained of 1 week history of shortness of breath.

Six years ago, patient had a saddle pulmonary embolus after a rotator cuff repair surgery. During that episode he had acute shortness of breath and pleuritic chest pain. A full hypercoagulable workup revealed protein C deficiency and hyperhomocysteinemia, but the workup was not considered definitive as patient was on coumadin at the time. He was treated with coumadin for one year and was lost to follow-up afterwards.

Upon arrival in ER, the initial physical examination revealed hypoxemia (pulse oximetry was in low-mid 80's) and arterial blood gas showed pH 7.44, PaCO_2 _33 mmHg, PaO_2 _42 mmHg on room air. His lower extremities were cold and pulseless from dorsal pedis to femoral arteries bilaterally. The blood pressure in the lower extremities was 87/44, while blood pressure in the upper extremities was 114/70, with a heart rate of 108 beats per minute. Neurological examination revealed intact cranial nerves, with preserved comprehension and speech. Muscle strength was normal in the upper extremities, but 4/5 in both lower extremities. Deep tendon reflexes were normal. Sensation was decreased in lower extremities. EKG showed sinus rhythm at 108 per minute. The patient was treated with intravenous heparin empirically for pulmonary embolism.

Within 2–3 hours after presentation, patient's symptoms improved with resolution of groin pain, and return of the sensation of his lower extremities.

**Figure 1:**
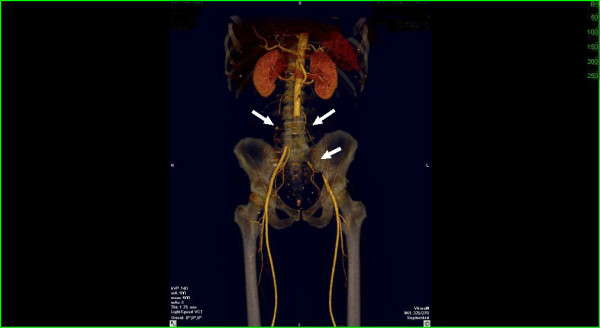
Aortogram of abdomen and pelvis shows occlusion in the abdominal aorta extending into bilateral common iliac arteries, internal and external iliac arteries.

CT aortogram of the abdomen and pelvis with lower extremity femoral runoff revealed occlusion in the abdominal aorta below the level of the renal arteries extending into the inferior mesenteric artery, occlusion in the distal aorta just above the bifurcation extending into bilateral common iliac arteries, internal and external iliac arteries. There was also occlusion seen in the distal right popliteal artery extending into the tibial arteries. On the left there is approximately a large 5.5 cm occlusion of the left internal iliac artery with reconstitution distally. A wedge shaped perfusion defect was also noted in the upper pole of the right kidney suggestive of a renal infarct. The renal arteries were both patent without significant stenosis. Given there was no evidence of atherosclerosis and the rapid onset nature of his symptoms, the filling defects were believed to be emboli (see figure 1).

**Figure 2:**
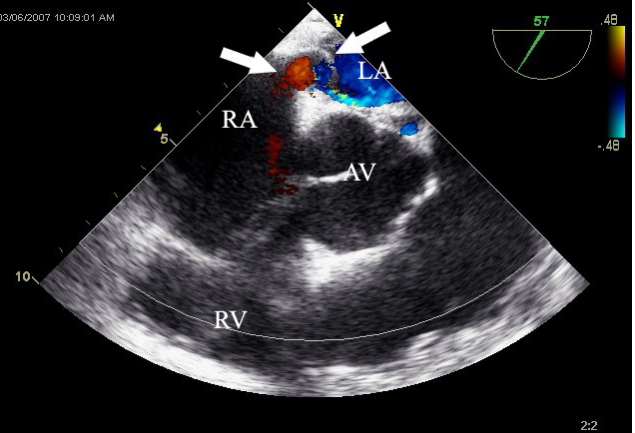
Echocardiogram shows the presence of a large PFO.

Emergent vascular surgery consult was requested and emergent thrombectomy and IVC filter insertion were scheduled. A transesophageal echo was done intraoperatively which revealed normal left ventricle with mildly depressed systolic function, with an estimated ejection fraction of 50%. There was flattening of the interventricular septum consistent with right ventricular pressure overload. The interatrial septum was mobile with a large PFO and a large right to left shunt identified by color Doppler analysis and agitated saline injection. The right ventricle was severely enlarged and hypokinetic. The right atrium was moderately enlarged. RV systolic pressure is 58 mmHg and the IVC was also dilated. There was mild-to-moderate tricuspid regurgitation with pulmonary hypertension. No other cardiac thrombi were noted (see figure 2).

An emergency IVC filter was placed. The patient underwent bilateral aortic embolectomies, bilateral iliac embolectomies, and bilateral superficial femoral profunda embolectomies, and bilateral tibial-peroneal trunk embolectomies via bilateral femoral arteriotomies.

Doppler ultrasound of the lower extremities done postoperatively revealed thrombosis in the left proximal superficial femoral vein and left popliteal vein extending into the posterior tibial vein.

Patient was transferred to cardiovascular step-down unit afterwards and subsequent anticoagulation with heparin and coumadin was resumed.

His anticoagulation work up showed no evidence of factor V Leiden or prothrombin gene G20210A mutation, antiphospholipid antibody screening was negative, and levels of factor VIII and homocysteine were normal. Levels of protein C, S and antithrombin III were unreliable in the setting of an acute thromboembolic event or during treatment with heparin and coumadin.

The patient was discharged 8 days after admission, no recurrent emboli have been observed. He is to stay on lifelong coumadin therapy. PFO closure was planned at a later date.

## Discussion

This patient most likely has a hypercoagulable state which predisposed him to DVT. Emboli from the venous system caused subacute PE and subsequent pulmonary hypertension which caused the right to left shunt through his PFO, leading to the formation of paradoxical embolism.

The incidence of paradoxical emboli is relatively low; fewer than 2% of all cases of systemic arterial emboli have been described. PFO has been reported to be an important risk factor for paradoxical systemic embolization because of right-to-left-shunting[3]. Paradoxical embolism is frequently associated with cryptogenic stroke as well as peripheral embolism, brain abscess, and decompression sickness in underwater divers [4]. Other clinical manifestations of paradoxical embolus that have been described include myocardial infarction [5], renal infarction, and retina artery occlusion [6].

The immediate recognition of paradoxical embolus is of utmost importance. Treatment mainly consists of thrombectomy or thrombolysis. With concomitant PE, a permanent IVC filter and anticoagulation are often needed.

Closure of the PFO after the first embolic event is suggested for patients at high risk for a recurrent embolic event. The risk factors associated with recurrence of embolism include atrial septal aneurysm, high shunting volume, and shunting at rest. Other characteristics implicated include a PFO larger than 3.4 mm (up to 5.8 mm in different studies), high mobility of the PFO valve, a well-developed Eustachian valve, a Valsalva maneuver immediately prior to the event, a history of recurrent embolic events, and perhaps a hypercoagulable state [7]. However there is no definite evidence that hypercoagulable states would increase the risk of embolic events in a patient with PFO. Surprisingly, one report indicated thrombophilia does not seem to be an additional factor to the excess of risk observed in young patients with cryptogenic stroke and PFO [8].

In our patient, recurrent embolic events, the large PFO plus his hypercoagulable state warrant closure of the PFO. A follow up TEE is planned to evaluate his RV function and subsequent PFO closure plan will be based on the results of TEE.

## Conclusion

In summary, paradoxical embolism and intracardiac shunt should be immediately considered in a patient with PE and systemic arterial embolism. Diagnostic modalities included Doppler ultrasound, CT angiogram, aortogram and saline contrast echocardiography. The treatment regimen in such cases includes thrombectomy, thrombolysis, an IVC filter (if indicated) and anticoagulation. If PFO closure is indicated, right ventricular function should be assessed pre-operatively to prevent precipitation or exacerbation of right ventricular dysfunction.

## Abbreviations

ABG = arterial blood gas; BP = blood pressure; DVT = deep venous thrombosis; EKG = electrocardiogram; IVC = inferior vena cava; PE = Pulmonary embolism; PFO = patent foramen ovale; RV = right ventricle; TEE = transesophageal echocardiogram.

## Competing interests

The author(s) declare that they have no competing interests.

## Authors' contributions

SG collected the data and drafted the manuscript. Both IR and JM revised and approved the final manuscript.
